# Tissue transglutaminase-induced alterations in extracellular matrix inhibit tumor invasion

**DOI:** 10.1186/1476-4598-4-33

**Published:** 2005-09-09

**Authors:** Lingegowda S Mangala, Banu Arun, Aysegul A Sahin, Kapil Mehta

**Affiliations:** 1 Department of Experimental Therapeutics, The University of Texas M. D. Anderson Cancer Center, Houston, Texas, USA; 2 Department of Breast Medical Oncology, The University of Texas M. D. Anderson Cancer Center, Houston, Texas, USA; 3 Department of Pathology, The University of Texas M. D. Anderson Cancer Center, Houston, Texas, USA; 4 Cancer Biology Program, Graduate School of Biomedical Sciences, The University of Texas, Houston, Texas, USA

## Abstract

**Background:**

Alterations in the extracellular matrix (ECM) can affect host-tumor interactions and tumor growth and metastasis. Tissue transglutaminase (TG2, EC 2.3.2.13), a calcium-dependent enzyme that catalyzes covalent cross-linking of proteins, can render the ECM highly stable and resistant to proteolytic degradation. So we determined whether TG2 expression in a tumor or nontumor (stroma) environment could affect the process of metastasis. Two hundred archived samples from patients with breast cancer were studied for the TG2 expression. Also, in an *in vitro *model the invasive behavior of MDA-MB-231 cells in the presence or absence of exogenous TG2 was determined.

**Results:**

Tumors associated with negative nodes showed significantly higher expression of TG2 in the stroma (*P < 0.001*). TG2 in the stroma was catalytically active, as revealed by the presence of isopeptide cross-links. Pretreatment of Matrigel with catalytically active TG2 resulted in strong inhibition of invasion of MDA-MB-231 cells through the Matrigel Transwell filters.

**Conclusion:**

TG2-induced alterations in the ECM could effectively inhibit the process of metastasis. Therefore, selective induction of catalytically active TG2 at the site of tumor may offer promising approach for limiting the metastasis.

## Background

Despite significant advances in the treatment of primary breast cancer, predicting and preventing metastasis remains a daunting clinical challenge. To make progress in this area, it is imperative to understand the molecular mechanisms that regulate the progression from a primary tumor to metastatic disease.

Metastasis is a multistep process that involves intravasation, adhesion to a blood vessel wall, extravasation, infiltration, and the proliferation of cancer cells in the target tissue [1]. Many of these steps require interaction between tumor cells and the extracellular matrix (ECM). For example, the ECM can modulate tumor cell growth by binding to and storing cytokines, it can promote cell attachment and migration by providing a stable foundation, and it can support cell growth and survival by interacting with cell-surface receptors and activating appropriate signaling pathways [2,3].

Several lines of evidence have suggested that tissue transglutaminase (TG2, EC 2.3.2.13) plays an important role in stabilizing the ECM by cross-linking its component proteins and rendering it resistant to mechanical and proteolytic degradation [4-7]. TG2, a member of the Ca^2+^-dependent family of mammalian enzymes, catalyzes irreversible cross-linking of proteins by inserting highly stable ε(γ-glutamyl)lysine bonds between them [5,8,9]. Several ECM proteins, such as fibronectin, vitronectin, collagen, fibrin, laminin, osteonectin, and osteopontin, can serve as substrates in TG2-catalyzed cross-linking reactions [4,10-12]. Moreover, in various fibrotic disorders, such as pulmonary fibrosis, renal fibrosis, and atherosclerosis increased expression of TG2 has been observed, and its ability to cross-link ECM proteins has been implicated in facilitating the deposition of a new ECM and making it resistant to metalloproteinases [12-16]. In addition to its direct role in promoting the accumulation of the ECM, TG2 has been implicated in the storage and activation of transforming growth factor-beta (TGF-β) [17], a proinflammatory cytokine that is involved in the synthesis of various ECM proteins and inhibitors of metalloproteinases [18,19]. The ability of TG2 to affect the physicochemical properties of the ECM may influence the invasive properties of tumor cells by modulating cell-matrix interactions or by facilitating the assembly of the matrix and tissue remodeling.

In view of these facts and other observations that modification of the ECM can affect the growth of both normal and cancerous mammary epithelial cells and the processes of angiogenesis and tumor metastasis [20-22], we speculated that TG2 expression in the stroma of the host can affect breast cancer progression. To test this theory, we searched for such a correlation in tumor and stroma specimens in a total 200 samples from patients with early-stage breast cancer. Our findings suggested that TG2 expression in the stroma was associated with an absence of lymph node metastasis in patients with breast cancer. The results of our *in vitro *study further supported this link and suggested that TG2-mediated modification of the ECM could render it less susceptible to invasion by tumor cells. Taken together, these findings suggest that TG2 is a good candidate for therapeutic use to prevent progression from a primary tumor to metastatic disease in patients with breast cancer.

## Results

Of the 200 samples studied, only 189 were evaluable (Table 1). Patients without lymph node metastasis (n = 95) were followed for a median of 4 years after diagnosis. Two of these patients experienced disease recurrence, and 4 died. Patients with lymph node metastasis (n = 94) were followed for a median of 3.2 years after diagnosis. Ten of these patients experienced disease recurrence, and 2 died.

**Table 1:** TG2 expression in tumor and stroma tissues of patients with breast cancer

		**TG2 in tumor**					**TG2 in stroma**				
		
	**Total**	**0**	**1+**	**2+**	**3+**	p*	**0**	**1+**	**2+**	**3+**	P*
**Tumor samples**	189	84	37	33	35		85	52	28	24	
**Node status**											
Negative	95	46(48)	17(18)	17(18)	15(16)		24(25)	33(35)	20(21)	18(19)	
Positive	94	38(41)	20(21)	16(17)	20(21)		61(65)	19(20)	8(9)	6(6)	
						0.28					<0.001
**Age (yrs)**											
<50	53	21(39)	11(21)	11(21)	10(19)		22(41)	11(21)	21(21)	9(17)	
>=50	136	63(46)	26(19)	22(16)	25(19)		63(46)	41(30)	17(13)	15(11)	
						0.51					0.14

* Cochran-Armitage trend test

The numbers in parenthesis indicate the percentages of total number of patients in that row.

**Figure 1:**
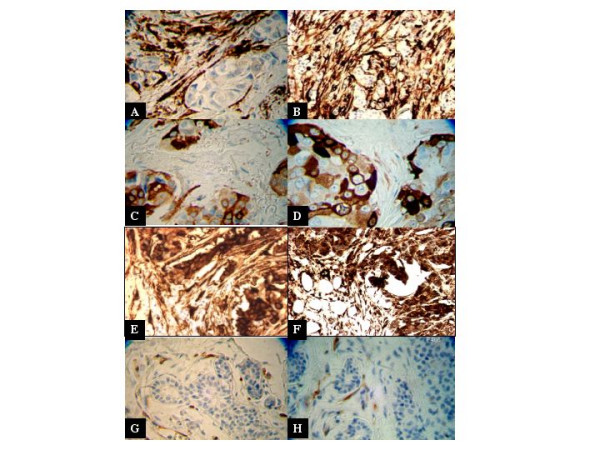
TG2 expression in tumor tissue samples from patients with breast cancer. Paraffin-embedded tissues from the primary tumors were retrieved from the Tumor Tissue Bank in the Department of Pathology at The University of Texas M. D. Anderson Cancer Center. The tissue sections were processed for immunostaining as described in Materials and Methods. One hundred samples each from patients with or without lymph node metastasis were studied and independently scored by a pathologist and a laboratory technician for TG2 expression. The figure shows the extent of TG2 expression in the primary tumor cells and the stroma surrounding the tumor from eight representative patients. *a *and *b*, tumor samples from representative patients (14% of patients) in which TG2 expression was mainly localized in the stroma; *c *and *d*, tumor samples from representative patients (23% of patients) in which TG2 expression was mainly localized in the tumor; *e *and *f*, TG2 expression in some patients' tumor samples (13% of patients) was observed both in the tumor and the stroma; *g *and *h*, half of all patients (50%) showed almost a complete lack of TG2 in the tumor and the stroma. The TG2-positive cells in *g *and *h *were endothelial cells that had high constitutive TG2 expression.

A typical pattern of TG2 expression in mammary tumor samples is shown in Fig. 1. Tumor samples showed either high TG2 expression that was predominantly restricted to the stroma surrounding the tumor (Fig. 1A &1B) or in some cases converse was true; tumors expressed high levels of TG2, but the stroma surrounding the tumor showed little or no TG2 expression (Fig. 1C and 1D). In few patient samples (approximately 12%) high TG2 expression was observed in both the tumor and the surrounding stroma (Fig. 1E and 1F). Similarly, some samples expressed little (≤1+) or no TG2 in the tumor or the stroma (Fig. 1G and 1H).

The association of TG2 expression in tumors and stroma with node-negative or -positive status is presented in Table 1. There was no evidence that TG2 expression in tumor cells differed between node-negative or node-positive samples. Nevertheless, there was strong evidence that tumors associated with negative nodes had a significantly higher TG2 expression in the stroma (*P *< 0.001). With regard to the association of TG2 expression in tumors and stroma with age, there was no evidence of a difference in younger (<50 yrs) or older (>50 yrs) patient populations.

**Figure 2:**
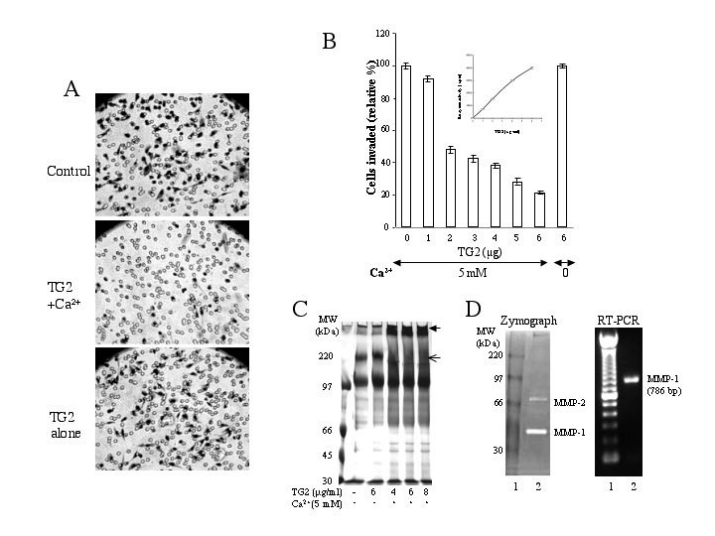
TG2 inhibits the invasion of MDA-MD-231 cells through a Matrigel-coated Transwell membrane. *A*, Matrigel contents were incubated with buffer alone (control) or buffer containing the purified guinea pig liver TG2 protein (6 μg/0.75 ml) in the presence (TG2 + Ca^2+^) or absence (TG2 alone) of 5 mM Ca^2+ ^at 37°C for 15 minutes before being coated onto the Transwell membranes. The MDA-MB-231 cells were compared for their ability to invade through the TG2-pretreated or untreated Matrigel-Transwell membranes. Representative fields with cells that migrated under the membrane were photographed. *B*, Matrigels (0.75 ml) containing increasing amounts of the purified TG2 protein were preincubated (37°C, 15 minutes) with 5 mM Ca^2+^. Another tube containing Matrigel plus 6 μg of TG2 protein was incubated in parallel, but without Ca^2+^, to serve as control. Matrigel contents pretreated with various amounts of TG2 were layered over the Transwell membranes and compared for their ability to support the invasion of MDA-MB-231 cells. Ten fields were counted randomly under the microscope for the number of cells that had migrated through the Matrigel and were plotted as an average number of cells ± SD per field. *C*, Equal volumes of Matrigel (0.75 ml) were incubated under conditions described in legend *B *with buffer alone or buffer containing varying concentrations of purified TG2 in the presence (+) or absence (-) of 5 mM Ca^2+^. Forty μl reactants, each were fractionated on SDS-PAGE. The gel was stained and viewed for TG2-induced changes in protein bands after destaining in methanol/acetic acid solution. The thin arrow indicates a prominent band that disappears in the presence of an enzymatically active TG2; whereas thick arrow indicates the appearance of a prominent band in the presence of active TG2. *D*, Basal levels of MMP-1 and MMP-2 in MDA-MB-231 cells as determined by zymogram performed on the supernatants collected from cultured cells (80% confluent in 6-well plate, incubated in 1 ml serum-free medium overnight) or by RT-PCR, using MMP-1 specific primers, as described in Materials and Methods.

To further delineate the importance of TG2 expression in the stroma and its possible implications in the modulation of metastatic disease, we used an *in vitro *Matrigel Transwell invasion assay with a human breast cancer cell line MDA-MB-231. Under our experimental conditions, MDA-MB-231 cells were highly invasive, as determined by the number of cells that invaded through the Matrigel Transwell filters (Fig. 2A). However, pretreatment of the Matrigel with catalytically active TG2 (in the presence of Ca^2+^) strongly inhibited the invasion of MDA-MB-231 cells through the Matrigel Transwell filters. Under identical conditions when the enzyme was rendered inactive by eliminating Ca^2+ ^from the reaction mixture, the number of cells that invaded was not much different from that seen in the control wells (coated with Matrigel that had been preincubated with 5-mM Ca^2+ ^but without TG2). TG2-mediated inhibition of tumor cell invasion was dose-dependent and could be observed by pretreating the Matrigel contents with as little as 1 μg of TG2. At 6 μg, TG2 inhibited the invasion of MDA-MB-231 cells by more than 80% (Fig. 2B). The inhibition of cell invasion through TG2-pretreated Matrigels was most likely due to the crosslinking of component proteins into high molecular weight scaffolds (Fig. 2C). The TG2-induced crosslinks are known to exhibit resistance to proteases and thus may pose a barrier against protease-induced invasion of cancer cells. Indeed, MDA-MB-231 cells produce active proteases, such as MMP-1 and MMP-2 as revealed by zymography and RT-PCR analysis (Fig. 2D). TG2 by itself, either in the presence or in absence of Ca^2+^, did not exert any noticeable effect on cell viability or growth of MDA-MB-231 cells (data not shown). These results suggest that active cross-linking of the Matrigel proteins by TG2 is effective in preventing the invasion of tumor cells.

**Figure 3:**
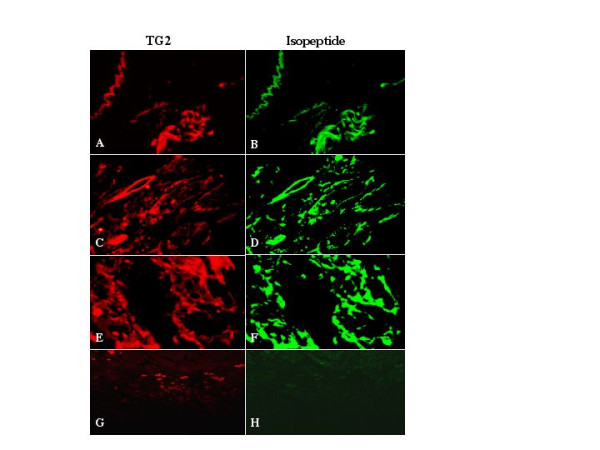
TG2 expression in the stroma is closely associated with the formation of isopeptide bonds. Tissue sections from 3 selected patients with breast cancer that showed high TG2 expression in the stroma were incubated simultaneously with rabbit anti-TG2 antibody and mouse anti-isopeptide monoclonal antibody. Anti-mouse IgG Alexa 488 and goat anti-rabbit IgG Alexa 546 were used as secondary antibodies to localize the presence of TG2 (A, C, E) and isopeptide (B, D, F), respectively. In a control experiment (G, H), a tissue sample lacking TG2 expression in the tumor and the stroma was used in a similar way (two primary and 2 secondary antibodies) to determine the specificity of the antigen-antibody reactions.

Next, we sought to determine whether TG2 in the stroma of node-negative patients with breast cancer was catalytically active. To accomplish this, we studied the presence of ε(γ-glutamyl)lysine isopeptide, the product of TG2-catalyzed protein cross-linking reactions, in a few selected samples. The results (Fig. 3) confirmed strong positivity of the stroma for TG2 expression in selected node-negative patient samples (red fluorescence). Importantly, the expression of TG2 in stroma was closely associated with the presence of isopeptide bonds, as revealed by strong immunofluorescence staining with the anti-isopeptide antibody (green fluorescence). The expression of TG2 in the stroma, in general, closely resembled the isopeptide staining (Fig. 3), suggesting that the two antigens closely associate in the stroma surrounding the tumor. These results suggest that, in some patients, TG2 can localize extracellularly and effectively cross-link ECM proteins, thereby rendering the ECM resistant to invasion by tumor cells.

## Discussion

Major findings to emerge from this study are: in some patients with breast cancer, tumor and nontumor (stroma) environments express increased levels of TG2; in the stroma, TG2 protein exists in a catalytically active configuration, leading to cross-linking of the ECM proteins; and TG2-catalyzed cross-linking of ECM proteins may mitigate the migration of cancer cells to distant sites.

TG2 is a multifunctional protein that can affect the ECM and its interaction with cells. Both the extracellular cross-linking and intracellular signaling activities of TG2 can considerably modulate cell-matrix interactions [7]. We previously observed that TG2 expression was up-regulated in drug-resistant and metastatic breast cancer cells and cell lines [23-28]. The increased expression of TG2 in drug-resistant and metastatic breast cancer cells was linked to their increased resistance to apoptosis, owing to the fact that TG2 in these cells closely associates with β integrins and thus may promote cell survival signaling [28-30]. The primary objective of this study was to determine whether higher levels of TG2 in breast cancer cells were associated with node involvement and development of aggressive tumors. We found no evidence that TG2 tumor staining differed between tumor samples associated with negative or positive node status. These results are in agreement with those of a previous study that revealed a negative correlation between TG2 transcript expression and nodal status in patients with breast cancer [31]. However, in another report, Jiang et al. observed that lymph node involvement and development of aggressive phenotype in breast cancer tumors was associated with increased expression of TG2 transcript and decreased levels of TG3 and TG7 transcript expression [32]. Similarly, another group has reported a marked increase in TG2 expression in the intraductal and invasive human breast cancer cells [33]. Taken together, these observations suggest that transglutaminases' repertoire is altered during breast cancer development and progression. Therefore, further studies are warranted with larger cohort of patients to determine the prognostic significance of transglutaminases in the progression and development of metastatic disease in patients with breast cancer.

More important, the results of our study suggest that TG2 expression in the stroma is strongly associated with node-negative status in patients with breast cancer. A similar increase in TG2 expression in the stroma surrounding the tumor was observed by Haroon et al. in subcutaneously implanted rat mammary tumors [34]. Treatment of these tumors with enzymatically active TG2 significantly delayed the tumor growth when compared with the tumors treated with catalytically inactive TG2 mutant. On the basis of these observations the authors concluded that TG2 might constitute a distinct part of the host response against growing tumor and by crosslinking the component proteins, it may stabilize the ECM and affect the tumor growth [34].

It is possible that local injury caused by the growing tumor may elicit host's response and induce cytokines production that in turn may promote wound healing and restrict the invasion of cells by producing new or stable ECM [35]. Indeed, TG2 is capable of cross-linking several constituent proteins in the ECM, which can render the ECM more resistant to proteases and mechanical disruptions [4-6]. TG2 can also enhance stability and strengthen the ECM by its ability to facilitate the activation of tumor growth factor-beta [17]. Thus, TG2-mediated alterations may render the ECM more amenable to tumor growth and resistant to proteases [36-38]. Indeed, our *in vitro *data shown in figure 2 clearly supported this contention and suggested that TG2-mediated crosslinking of component proteins rendered the matrigel resistant to invasion by MDA-MB-231 cells regardless of the fact that these cells actively produced matrix metalloproteinases (MMP) such as MMP-1 and MMP-2.

In conclusion, our findings suggest that increased expression of TG2 in the stroma represents a part of the host response to a growing tumor in an attempt to restrict tumor growth and prevent it from spreading to distant sites. However, the ability of tumor cells to produce proteases and other factors that could render TG2 inactive may overwhelm the ability of TG2 to inhibit growth and prevent metastasis. Indeed, TG2 has been shown to be a good substrate for MMP-2-like proteases that are abundantly produced by metastatic tumors [39,40]. Future studies to selectively enhance the production of active TG2 at the site of tumor growth may offer promising approaches to limiting tumor growth and metastasis.

## Methods

### Cell Lines and Tumor Tissues

Human breast cancer cell line MDA-MB-231 was purchased from American Type Culture Collection (Rockville, MD) and cultured by standard methods. Tumor samples from 200 patients with early-stage breast cancer diagnosed between 1979 and 2002 were obtained from the archives of the Pathology Department of The University of Texas M. D. Anderson Cancer Center after approval by the Institutional Review Board for the Welfare of Human Subjects. All the selected patients had tumors ranging from 1 cm to 2 cm in largest diameter. Samples were selected for inclusion to yield 100 tumor samples that were associated with lymph node invasion and 100 tumor samples that were not associated with lymph node invasion.

### Invasion Assay

The invasive behavior of MDA-MB-231 cells in the presence or absence of exogenous TG2 was determined *in vitro *by counting how many cells invaded through Matrigel-coated Transwell polycarbonate membrane inserts, as described previously [41,42]. Briefly, Transwell inserts with a pore size of 12 μm were coated with 0.78 mg/ml Matrigel in cold, serum-free medium. In some cases, the Matrigel was preincubated (37°C for 15 minutes) with varying concentrations of purified TG2 protein (Sigma-Aldrich Chemical Co., St. Louis, MO) in the presence or absence of 5 mM Ca^2+ ^before being coated onto the inserts. Also, Matrigel samples (40 μl aliquots) pretreated with varying concentrations of TG2 in the presence or absence of Ca^2+ ^were subjected to 7% SDS-PAGE. The gel was stained with Coomassie brilliant blue and destained in a acetic acid and methanol solution before visualizing the changes in protein bands as a consequence of TG2-mediated crosslinking.

Cells were recovered by trypsinization and washed once with serum-free medium. The cell pellets were resuspended in serum-free medium, and 0.5 ml of the cell suspension (0.5 × 10^6 ^cells) was added to duplicate wells. After incubation for 48 hours, the cells that passed through the filter were stained using a Hema-3 stain kit (Fisher Scientific, Houston, TX), and the cells in 10 random fields were counted under a microscope.

### Matrix Metalloproteinases (MMP)

Basal expression of MMP-1 and MMP-2 in MDA-MB-231 cells was determined by gelatin-substrate gel zymography as described [41]. Briefly, the supernatant from MDA-MB-231 cultured cells was subjected under nonreducing conditions to gelatin impregnated (0.1%; w/v) SDS-PAGE. The gel was washed several times to remove the SDS and incubated in buffer containing 5 mM CaCl_2 _plus 1 μM ZnCl_2 _for 24–48 h at 37°C. The gel was stained with Coomassie brilliant blue and destained. Proteolytic activity was visualized as clear bands (zones of gelatin degradation) against the blue background of stained gelatin.

For RT-PCR, total RNA was isolated from MDA-MB-231 cells in Trizol reagent (Invitrogen, Life technologies, CA) and cDNA was synthesized from 5 μg of total RNA using Superscript reverse transcriptase (Life Technologies, Inc) as per the manufacturer's instructions. cDNA was subjected to PCR (GeneAmp PCR System 9700, Applied Biosystems) using MMP-1 specific, 5'-CGACTCTAGAAACACAAGAGCAAGA-3' (sense) and 5'-AAGGTTAGCTTACTGTCACACGCTT-3'(antisense) primers. PCR consisted of 30 cycles was carried out at the following conditions: 95°C for 5 min, 30 cycles of 95°C for 30 sec, annealing at 58°C for 1 min and elongation at 72°C for 2 min. PCR products were analyzed on 1% agarose gel and visualized under UV light after staining with ethidium bromide.

### Immunohistochemistry

Sections of formalin-fixed, paraffin-embedded tumor samples (5 μm thick) were heated to 60°C and dehydrated in xylene and graded alcohols. Antigen retrieval was performed with 0.01 M citrate buffer at pH 6.0 for 20 minutes in a 95°C steam bath. Slides were allowed to cool for 20 minutes at room temperature, followed by repeated rinsing with 0.1 M phosphate-buffered saline (PBS, pH 7.4) containing 0.1% Tween 20 (PBS-T). Endogenous peroxidase activity was quenched with 3% hydrogen peroxidase. Each incubation step was conducted at room temperature and was followed by three sequential washes (5–10 minutes each) in PBS-T. Sections were incubated with anti-TG2 monoclonal antibodies, CUB-7401 (Neomarkers, Fremont, CA) overnight at 4°C, followed by incubation for 30 minutes each with biotinylated secondary antibody and peroxidase-labeled streptavidin. Antigen-antibody reactions were detected by exposure to 3,3'diaminobenzidine and hydrogen peroxide chromogen substrate (Vector Labs, Burlingame, CA) for 3–5 minutes. Slides were counterstained with hematoxylin and mounted. The negative controls were incubated with nonimmune mouse immunoglobulin g (IgG) in place of the primary antibody. The immunostained slides were examined under the light microscope and scored (with values of 0, 1+, 2+, or 3+) independently by two researchers in the laboratory.

### Confocal Microscopy

Paraffin-embedded tissue sections from selected tumor samples were deparaffinized, washed 3 times with PBS, and blocked with 5% normal goat serum in PBS for 1 hour. The sections were immunostained by an indirect method using primary rabbit anti-TG2 (Neomarkers, Fremont, CA) and anti-isopeptide monoclonal Ab422 (Abcam, Cambridge, MA) antibodies. The anti-mouse IgG Alexa 488 and goat anti-rabbit IgG Alexa 546 (both from Molecular Probes, Eugene, OR) were used as the secondary antibodies. The immunostained sections were mounted in 80% glycerol and 20% PBS and viewed under the Zeiss Laser Scanning Microscope 510 (Carl Zeiss MicroImaging, Inc., Thornwood, NY) for taking the digital images. Appropriate controls, including mouse and rabbit IgG in place of the primary antibodies and either primary antibody alone along with both the secondary antibodies were included to determine the specificity of the reaction.

## Abbreviations

BCL2, B-cell leukemia/lymphoma 2; ECM, extracellular matrix; EGFR, epidermal growth factor receptor; FAK, focal adhesion kinase; Fn, fibronectin; MDR, multidrug resistance; TG2, tissue transglutaminase; TGF-β, transforming growth factor-beta.

### Authors' contributions

SLM, conducted the experiments. BA, identified and retrieved the patients samples. AS, evaluated the slides for TG2 expression, KM. Participated in the study design, data interpretation, and manuscript preparation.
